# The Association between the Perceived Adequacy of Workplace Infection Control Procedures and Personal Protective Equipment with Mental Health Symptoms: A Cross-sectional Survey of Canadian Health-care Workers during the COVID-19 Pandemic: L’association entre le caractère adéquat perçu des procédures de contrôle des infections au travail et de l’équipement de protection personnel pour les symptômes de santé mentale. Un sondage transversal des travailleurs de la santé canadiens durant la pandémie COVID-19

**DOI:** 10.1177/0706743720961729

**Published:** 2020-09-21

**Authors:** Peter M. Smith, John Oudyk, Guy Potter, Cameron Mustard

**Affiliations:** 1 30678 Institute for Work & Health, Toronto, Ontario, Canada; 2 Dalla Lana School of Public Health, University of Toronto, Ontario, Canada; 3 Department of Epidemiology and Preventive Medicine, Monash University, Melbourne, Australia; 4 Occupational Health Clinics for Ontario Workers, Hamilton, Ontario, Canada; 5 Department of Psychiatry and Behavioral Sciences, 22957 Duke University Medical Center, Durham, NC, USA

**Keywords:** occupational health, mental health, workplace safety, COVID-19

## Abstract

**Objectives:**

To examine the relationship between perceived adequacy of personal protective equipment (PPE) and workplace-based infection control procedures (ICP) and mental health symptoms among a sample of health-care workers in Canada within the context of the current COVID-19 pandemic.

**Methods:**

A convenience-based internet survey of health-care workers in Canada was facilitated through various labor organizations between April 7 and May 13, 2020. A total of 7,298 respondents started the survey, of which 5,988 reported information on the main exposures and outcomes. Anxiety symptoms were assessed using the Generalized Anxiety Disorder (GAD-2) screener, and depression symptoms using the Patient Health Questionnaire (PHQ-2) screener. We assessed the perceived need and adequacy of 8 types of PPE and 10 different ICP. Regression analyses examined the proportion of GAD-2 and PHQ-2 scores of 3 and higher across levels of PPE and ICP, adjusted for a range of demographic, occupation, workplace, and COVID-19-specific measures.

**Results:**

A total of 54.8% (95% confidence interval [CI], 53.5% to 56.1%) of the sample had GAD-2 scores of 3 and higher, and 42.3% (95% CI, 41.0% to 43.6%) of the sample had PHQ-2 scores of 3 and higher. Absolute differences of 18% (95% CI, 12% to 23%) and 17% (95% CI, 12% to 22%) were observed in the prevalence of GAD-2 scores of 3 and higher between workers whose perceived PPE needs and ICP needs were met compared to those who needs were not met. Differences of between 11% (95% CI, 6% to 17%) and 19% (95% CI, 14% to 24%) were observed in PHQ-2 scores of 3 and higher across these same PPE and ICP categories.

**Conclusions:**

Our results suggest strengthening employer-based infection control strategies likely has important implications for the mental health symptoms among health-care workers in Canada.

## Abstract (fr)

**Objectifs:**

Examiner la relation entre le caractère adéquat perçu de l’équipement de protection personnel (EPP) et les procédures de contrôle des infections (PCI) en milieu de travail et les symptômes de santé mentale au sein d’un échantillon de travailleurs de la santé du Canada, dans le contexte de la pandémie COVID-19 en cours.

**Méthodes:**

Un sondage de commodité sur Internet des travailleurs de la santé du Canada auquel ont collaboré diverses organisations professionnelles entre le 7 avril et le 13 mai 2020. En tout, 7 298 répondants ont commencé le sondage, et 5 988 d’entre eux ont donné de l’information sur les expositions principales et les résultats. Les symptômes anxieux étaient évalués à l’aide du dépisteur du trouble d’anxiété généralisée (GAD-2), et les symptômes de dépression, à l’aide du questionnaire sur la santé du patient (PHQ-2). Nous avons évalué le besoin et le caractère adéquat perçus de 8 types d’EPP, et de 10 PCI différentes. Des analyses de régression ont examiné la proportion des scores au GAD-2 et au PHQ-2 de 3 et plus élevés pour les niveaux d’EPP et de PCI, ajustés pour une série de données démographiques, d’occupations, de milieux de travail, et de mesures spécifiques pour la COVID-19.

**Résultats:**

Un total de 54,8% (intervalle de confiance à 95% 53,5% à 56,1%) de l’échantillon avait des scores au GAD-2 de trois et plus, et 42,3% (IC à 95% 41,0% à 43,6%) de l’échantillon avait des scores au PHQ-2 de trois et plus. Des différences absolues de 18% (IC à 95% 12% à 23%) et de 17% (IC à 95% 12% à 22%) ont été observées dans la prévalence des scores au GAD-2 de trois et plus entre les travailleurs qui percevaient que leurs besoins d’EPP et de PCI étaient comblés, comparé à ceux dont les besoins n’étaient pas comblés. Des différences entre 11% (IC à 95% 6% à 17%) et 19% (IC à 95% 14% à 24%) ont été observées dans les scores au PHQ-2 de trois et plus dans les mêmes catégories d’EPP et de PCI.

**Conclusions:**

Nos résultats suggèrent qu’un resserrement des stratégies de contrôle des infections par l’employeur a probablement des implications importantes pour les symptômes de santé mentale chez les travailleurs de la santé du Canada.

The COVID-19 pandemic is having profound impacts on workers across the globe.^1^ Based on previous outbreaks, such as SARS, it is recognized that health-care workers will be one of the occupational groups at highest risk of disease transmission.^2,3^ This increased risk along with concomitant increases in workload and fear of infecting family and household members will likely be associated with poorer mental health in this population.^4,5^

Within a conceptual model of occupational health and safety vulnerability,^6^ the combination of hazards exposure and inadequacy of protections at the workplace level will be associated with increased risk of injury and illness.^7^ A recent systematic review documented that distrust in infection control procedures (ICP) and inadequate provision of personal protective equipment (PPE) was associated with worse mental health among health-care workers.^5^ Pathways linking inadequate ICP and PPE to mental health within a health-care setting include lack of control over personal risk of infection, and associated increases in the risk of infection of family and other household members, as well as increased workload due to increased infection among coworkers and patients.^5,8,9^

The objective of this study is to examine the relationship between perceived adequacy of PPE and workplace-based ICP and anxiety and depression symptoms among a large sample of health-care workers in Canada, within the context of the current COVID-19 pandemic. We hypothesize that increased adequacy of both PPE and ICP is associated with a lower severity of anxiety and depressive symptoms. PPE and ICP sit in different locations on the hierarchy of controls, with PPE referring to items worn by workers to prevent infection, while ICP refers to changes in how workers perform their duties or the use of engineering controls in the workplace.^10^ As such, it is of interest to understand whether there is an interaction present in the association between PPE and ICP and symptoms of anxiety or depression, or whether the associations between PPE and ICP, and symptoms of anxiety or depression are additive.

## Methods

In April 2020, an online survey was developed by the Occupational Health Clinic for Ontario Workers with input from the members of an ad hoc pandemic survey group consisting of union health and safety representatives, activists, and academics. The survey was disseminated to health-care workers across Canada via various labor organizations. The current article focuses on responses to the survey between April 7, 2020, and May 13, 2020. The survey was not limited to particular groups of health-care workers or particular types of health-care settings. The survey was available in English and French. A total of 7,833 respondents opened the survey link, of which 7,298 answered at least 1 question. The convenience-based sampling precludes estimating a response rate. However, based on the Labour Force Survey, approximately 965,686 persons were employed in health-care occupations in the health and social assistance sector in Canada during this time period,^11^ which would suggest our sample represents 7.6% of the entire target population.

### Main Outcomes: Symptoms of Anxiety and Depression

Respondents were asked questions from the Generalized Anxiety Disorder (GAD-2) screener^12^ and the Patient Health Questionnaire (PHQ-2) screener.^13^ The range of possible scores for each scale is between 0 and 6. Previous studies have observed that a cut point of 3 or greater on the GAD-2 has a likelihood ratio of 5.0 for generalized anxiety disorder and 5.2 for any anxiety disorder.^12^ For the PHQ-2, a cut point of 3 or greater had a likelihood ratio of 2.9 for predicting major depression.^13^ Both measures were developed to screen for potential clinical disorders and to estimate symptom severity in health surveys; however, neither is diagnostic as a standalone measure.

### Main Independent Variables: Perceived Adequacy of PPE and Workplace ICP to Reduce COVID-19 Transmission

Respondents were asked questions on their perceptions of the adequacy and supply of 8 different types of PPE and the adequacy of implementation of 10 different ICP to reduce COVID-19 transmission.

PPE items included gloves, eye protection/goggles, face shields, gowns, hand sanitizer, surgical/procedural masks, N95 masks, and powered air particular respirators (PAPRs). Response options were appropriate type and adequate supply; appropriate type, but inadequate supply; inappropriate type, but adequate supply; inappropriate type and inadequate supply; needed, but not available at all; not sure/don’t know what is appropriate; and not applicable. We defined the need for a given PPE as when respondents endorsed one of the first 5 categories (compared to not sure/don’t know and not applicable). We defined that need being met when respondents endorsed the first category (appropriate type and adequate supply).

ICP items included screening of incoming patients, having symptomatic patients wearing masks, keeping patients with respiratory symptoms isolated from other patients and staff, restricted access and controlled flow of COVID patients though the facility, ventilation systems, airborne infection isolation rooms, personal hygiene facilities/locker rooms, house cleaning/disinfection practices, laundry cleaning practices, and waste disposal practices. Response options were appropriate and adequately implemented, appropriate but inadequately implemented, inappropriate, lacking, not sure/don’t know what is appropriate, and not applicable. We defined the need for a given ICP as when respondents endorsed one of the first 4 categories (compared to not sure/don’t know and not applicable). We defined that need being met when respondents endorsed the first category (appropriate and adequately implemented).

Respondents were grouped based on the proportion of their PPE and ICP needs that were met: those who had all their PPE and ICP needs met, those who had over half of their PPE and ICP needs met, those who had less than half of their PPE and ICP needs met, and those who did not have any of their PPE or ICP needs met.

### Covariates

Information was also collected on respondent age, gender, whether they identified as a visible minority, their province of residence, and whether they lived in an urban, suburban, or rural community. Information was also collected on the type of health-care facility in which they worked, their current job tenure, and their current hours of work per week. COVID-19-related exposures included how much contact respondents had with COVID-19 patients, the number of patients and workers at their workplace who have been infected with COVID-19 (suspected, presumed, or confirmed), whether they had experienced COVID-19 symptoms, and whether they had received training in relation to COVID-19 and in the donning and doffing of PPE.

### Analyses

The original sample of respondents who opened the survey and passed through the informed consent and answered at least 1 question totaled 7,298. Of this sample, 341 (4.7% of the sample) did not respond to the 2 GAD-2 questions, with another 39 (0.5% of the sample) not responding to the 2 PHQ-2 questions. As these questions were asked at the start of the survey, most of these respondents did not answer any further questions, leaving a sample of 6,918. Of this sample, 808 respondents (11.1% of the sample) did not respond to the questions on PPE, with another 122 additionally not responding to questions on ICP. This left a sample of 5,988 respondents (82.1% of the initial sample) with complete information on our outcomes and main independent variables. We compared scores for our 2 mental health outcomes between respondents with information on the main exposure compared to those with missing information. We did not observe any statistical differences across groups in relation to GAD-2 scores but did observe a slight trend for respondents with missing information on our independent variables to have lower scores on the PHQ-2 than respondents who were not missing information.

Where respondents were missing information on covariates, we defined a separate level of that variable for those with missing information to maximize the sample available.^14^ The size of missingness across covariates varied from less than 1% for measures describing the numbers of patients and workers with COVID-19, and contact with COVID-19 patients, to approximately 14% of the sample for information on the type of facility where they were employed.

Initial analyses examined the distributions of outcomes, independent variables, and covariates. To examine the relationship between perceived adequacy of PPE and ICP with symptoms of anxiety and depression, we ran separate regression models. Models were run examining the adjusted prevalence of respondents with GAD-2 and PHQ-2 scores of 3 and higher. We also ran separate regression models where the GAD-2 and PHQ-2 scores were included as continuous outcomes, and log-binomial models examining the relative risk of having GAD-2 and PHQ-2 scores of 3 and higher (given the prevalence of these outcomes in our data). The relationship between PPE and ICP adequacy and the outcomes in each of these models was similar, so only adjusted prevalence estimates are reported in this article. Interaction between PPE and ICP was examined by including an interaction term in the regression model. Regression models were examined for multicollinearity between measures. Slight collinearity between variables for missing responses to province and geographical density was noted; however, the relationships between the independent variable and outcomes did not change when one of these variables were omitted, compared to when both were included in the model. Models were run using PROC SURVEYREG in SAS Version 9.4,^15^ with the variance around each proportion estimated using a Jackknife estimation procedure.

## Results

**Figure 1.**
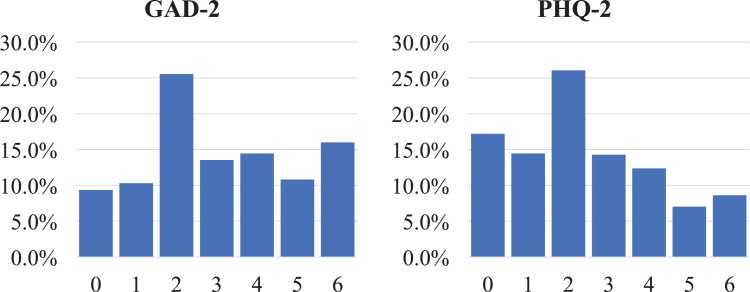
Distribution of Generalized Anxiety Disorder (GAD-2) and Patient Health Questionnaire (PHQ-2) scores among Canadian health-care workers (*N* = 5,988).

The distribution of the GAD-2 and PHQ-2 scores across the sample is presented in Figure 1. We observed respondents in every possible level of both measures. A total of 54.8% (53.5% to 56.1%) of the sample had GAD-2 scores of 3 and higher, and 42.3% (41.0% to 43.6%) of the sample had PHQ-2 scores of 3 and higher.

Table 1 presents the responses to questions on PPE and ICP. Respondents reported needing most types of PPE, with the exception of PAPRs. Unmet needs were highest for N95 masks and surgical procedural masks, followed by face shields, eye protection, and gowns. Self-reported needs for ICP related to COVID-19 transmission were generally lower than for PPE, with the highest needs for screening incoming patients and cleaning and disinfecting practices. The highest unmet needs were for personal hygiene and locker facilities, followed by screening of patients, having symptomatic patients wearing masks, and cleaning and disinfecting practices.

**Table 1.** Reported PPE and ICP Needs and Proportion Needs Being Met among Canadian Health-care Workers (*N* = 5,988) Related to COVID-19 Infection.

PPE	% Needed	% with Needs Met^a^	ICP	% Needed	% with Needs Met^a^
Gloves	96.9	79.8	Screening of patients	89.9	56.3
Eye protection	85.6	52.2	Symptomatic patients wearing masks	79.2	49.9
Face shields	85.7	42.9	Keeping patients with respiratory symptoms isolated	73.9	55.0
Gowns	90.8	56.6	Restricted access of COVID patients	70.2	61.4
Hand sanitizer	99.3	70.1	Ventilation systems	47.3	45.3
Surgical/procedure masks	95.3	42.7	Airborne infection isolation rooms	57.2	37.3
N95 masks	84.8	29.1	Personal hygiene facilities	79.0	32.2
PAPRs	21.5	18.6	Cleaning/disinfection practices	88.7	55.4
			Laundry cleaning	67.6	64.9
			Waste disposal	73.4	66.4

*Note*. PPE = personal protective equipment; ICP = infection control procedures; PAPRs = powered air particular respirators.

^a^ Proportion only of those who have need.

Descriptive information on the sample is provided in Table A1 in the Online Appendix of this article. The majority of respondents were female (91%), worked in hospitals (67%), were from the province of Ontario (56%), from urban centers (59%), and were working between 30 and 54 hours per week (75%). Experiences with COVID-19 differed across the sample with 39% of the sample having direct contact or working within 6 feet of COVID-19 patients, almost 60% reporting that 1 or more patients in their workplace have COVID-19, one-third reporting that their coworkers may have COVID-19, and just over one-fifth personally experiencing COVID-19 symptoms.

Table 2 presents the distribution of the proportion of PPE and ICP needs that were met and the adjusted proportion of respondents who had GAD-2 and PHQ-2 scores of 3 and higher. Although unmet needs were more common for ICP than for PPE, less than 20% of the sample had all of their PPE needs met or all their ICP needs met. After adjustment for study covariates, we observed a graded relationship between the proportion of PPE and ICP needs that were not met with an increasing proportion of respondents having GAD-2 and PHQ-2 scores of 3 and higher. Among the sample with 100% of their PPE needs met, 43% had GAD-2 scores of 3 or higher. This increased to 60% for those with none of their needs met; an absolute difference of 17.6% (12.1% to 23.1%). Similar relationships were observed for ICP needs and for PHQ-2 scores over 3. Comparisons of proportions indicated statistically significant differences across all levels of PPE and ICP needs being met for both outcomes with 2 exceptions. These were for differences in the proportion of PHQ-2 scores of 3 and higher for respondents with 1% to 49% of PPE needs met compared to those with none of their PPE needs met and for respondents with 100% of their ICP needs met and respondents with 50% to 99% of their needs met. We conducted a test for the trend in prevalence for each outcome across exposure groups. Apart from the trend for PPE needs and GAD-2 scores, which was linear, all other trend analyses indicated a nonconstant difference between the prevalence of the outcome across levels of the exposure, with the largest increases in prevalence generally seen when moving from 50% to 99% of needs met to 1% to 49% of needs met.

**Table 2.** Adjusted^a^ Prevalence of Respondents with GAD-2 and PHQ-2 Scores of 3 and Higher by PPE and ICP Needs Being Met.

Independent Variable	*N* (%)	GAD-2 Score 3 and Higher		PHQ-2 Score 3 and Higher	
		Prevalence %^b^	95% CI	Prevalence %^b^	95% CI
PPE needs					
100% of needs met	1,098 (18.3%)	42.9	28.4 to 57.3	33.2	22.2 to 44.2
50% to 99% of needs met	2,235 (37.3%)	49.6	35.1 to 64.1	37.5	26.5 to 48.4
1% to 49% of needs met	2,187 (36.5%)	55.5	40.8 to 69.7	42.2	31.2 to 53.1
None of needs met	468 (7.8%)	60.4	45.7 to 75.1	44.5	33.1 to 52.8
ICP needs					
100% of needs met	1,011 (16.9%)	43.4	28.9 to 57.9	30.1	19.3 to 40.9
50% to 99% of needs met	2,434 (40.7%)	47.5	33.1 to 61.8	33.4	22.5 to 44.2
1% to 49% of needs met	1,696 (28.3%)	56.6	42.2 to 71.1	44.7	33.7 to 55.6
None of needs met	847 (14.1%)	60.6	46.1 to 75.2	49.2	37.9 to 60.4

*Note*. *N* = 5,988. PPE = personal protective equipment; ICP = infection control procedures; GAD-2 = Generalized Anxiety Disorder; PHQ-2 = Patient Health Questionnaire.

^a^ Adjusted for age, sex, visible minority status, province of residence, population density, type of health-care facility, date when survey was completed, job tenure, current work hours, interactions with COVID-19 patients, patients at workplace with COVID-19, coworkers at workplace with COVID-19, experiencing COVID-19 symptoms, training related to COVID-19, and training in donning and doffing PPE.

^b^ All prevalence estimates in the table are statistically different from each other (as assessed using a 2-sided pairwise least squares mean difference test), with the exception of respondents with 1% to 49% of PPE needs met and those with none of their PPE needs met, and respondents with 100% of their ICP needs met and respondents with 50 to 99% of their needs met, when examining PHQ-2 scores of 3 and higher.

Other variables associated with anxiety symptoms in our regression models were having PPE training needs not met (compared to met), working in a long-term care facility (compared to a hospital), identifying as female, younger age, having COVID-19 symptoms, being in direct contact with COVID-19 patients (compared to no contact), not knowing how many coworkers in the facility have COVID-19 (compared to no coworkers having COVID-19), not knowing how many patients in the facility have COVID-19, and knowing 20 or more patients who have COVID-19 (compared to knowing no patients having COVID-19). Respondents from British Columbia had lower prevalence of anxiety symptoms compared to respondents from Ontario. Similar relationships were observed in regression models where PHQ-2 was the outcome. The variance in GAD-2 scores and PHQ-2 scores explained by all variables in the regression model was 14% and 12%, respectively. Exact estimates for all variables are available from the authors on request.

We did not observe any interactive relationship between adequacy of PPE and ICP with either outcome. As such, risks for PPE and ICP can be considered additive. To put this in context, among respondents who had adequate PPE and ICP, the prevalence of GAD-2 scores of 3 and higher was 34.8% (19.9% to 49.8%), while among respondents who had none of their PPE and ICP needs met, the prevalence of GAD-2 scores of 3 and higher was 71.6% (56.1% to 87.0%). This equates to an absolute difference of 36.7% (28.8% to 44.6%). For PHQ-2 scores of 3 and higher, the prevalence was 24.9% (13.5% to 36.2%) among respondents with adequate PPE and ICP, 54.4% (41.3% to 67.5%) for respondents with none of their PPE and ICP needs met, and 29.6% (21.1% to 38.1%) absolute difference in the prevalence between these groups.

## Discussion

The COVID-19 pandemic is placing increasing strain on health-care workers globally. Understanding and addressing the mental health among the health-care workforce is an essential component of the response and management of COVID-19 at the population level.^1,4^ Within this context, it is important to identify modifiable workplace factors that are associated with mental health outcomes.^5^ In this sample of almost 6,000 health-care workers in Canada, we observed a graded relationship between the perceived adequacy of PPE provision and ICP implementation with symptoms of anxiety and depression, with lower symptoms among respondents whose PPE and ICP needs were being met. These findings are consistent with a recent systematic review that suggested that employer-based infection control strategies were an important component to minimize psychological ill-health among health-care workers.^5^

We observed high prevalence rates of anxiety and depression symptoms in our sample (55% screened positive for anxiety symptoms and 42% for depression symptoms). The review mentioned above^5^ identified 7 quantitative studies that have examined levels of mental health distress among health-care workers during the current COVID-19 pandemic (2 published and 5 in preprint), all among Chinese health-care workers.^16–22^ Two of these studies used the GAD-7 and PHQ-9 survey instruments to assess anxiety and depression,^16,19^ which are somewhat comparable to our study as the GAD-2 and PHQ-2 consist of the first 2 items on these instruments. In these 2 studies, 14% to 15% of the respondents had PHQ-9 scores of 10 and higher. The cut point for the GAD-7 differed in these studies from greater than or equal to 7, and greater than or equal to 10, with proportions of anxiety also differing from 24% to 12%, respectively.^16,19^ Although direct comparability between the GAD-2 and GAD-7, and the PHQ-2 and PHQ-9 is hampered by the different response distributions for the various questions,^23^ it would appear that levels of anxiety and depression symptoms in our sample (55% and 42%) are considerably higher than those reported in these Chinese samples. Some of this difference may be due to cultural factors. For example, recent studies from the United Kingdom and Ireland have reported levels of depression symptoms (using the PHQ-9 score of 10 or more) and anxiety symptoms (using the GAD-7 score of 10 or more) during the COVID-19 pandemic are between 20% and 22% in general population samples.^24,25^ Given that we might expect higher levels among health-care workers, the levels of depression and anxiety symptoms reported in our sample are plausible. As such, greater surveillance and action concerning the mental health of health-care workers during and post the COVID-19 pandemic appear warranted as does the ongoing assessment of anxiety and depression symptoms.

The results of this study should be interpreted considering the following strengths and limitations. First, the GAD-2 and PHQ-2 were developed for screening purposes and are not equivalent to formal clinical diagnoses for anxiety and depression. Our sample was convenience-based, with survey respondents primarily made aware of the survey through labor organizations. In addition, participation across some provinces, in particular Quebec where COVID-19 cases were highest during the study period, was low, despite the survey being available in both official languages. Our nonresponse analysis indicated that respondents with missing data on our exposures had slightly lower PHQ-2 scores than those with complete information. As such, we suggest caution in generalizing the prevalence of mental health conditions in our sample to all health-care workers in Canada, in particular those in Quebec. To better understand the potential for selection bias, we compared the distribution of age, gender, and job tenure in our sample to estimates from health-care workers in Canada from the March and April 2020 Labour Force Surveys.^11^ We noted similar distributions in our sample to this representative sample of health-care labor force participants. Our sample had slightly fewer respondents aged 18 to 24 years (4% vs. 7%), and slightly more respondents aged 35 to 44 years (28% vs. 25%), a higher proportion of women (91% vs. 84%), a lower proportion of respondents with job tenure of 2 years and less (16% vs. 23%), and fewer part-time workers (15% vs. 25%). We do not believe that participation through labor organizations would significantly bias our sample as 73% of health-care workers in Canada are members of a union or have a collective bargaining agreement.^11^ Finally, given we have respondents across all levels of our exposures and all levels of our outcomes, the relationships we have observed between exposures and outcomes are likely still valid.^26,27^ The results in this article only reflect the experiences of health-care workers between April 7 and May 13, 2020. The number of COVID-19 cases and the number of hospitalizations have reduced considerably since the end of our survey. For example, the average number of COVID-19 cases per day in Canada during our study period was 1,520, while between June 13 (1 month after our survey) and the end of June, the average number of cases per day in Canada was 357.^28^ It is also likely that there have been concurrent changes in the adequacy of PPE and ICP in health-care settings. The ongoing assessment of PPE and ICP adequacy, and the mental health of health-care workers, is warranted to better understand these changes. Our exposure was based on perceived need for, and subsequent adequacy of, PPE and ICP. It is possible that our observed associations are primarily due to greater perceived numbers of PPE or ICP needs, as more overall needs could be associated with more unmet needs. In addition, it is possible that respondents who report more symptoms of anxiety and depression were more likely to perceive greater PPE and ICP needs. To examine this possibility, we did run regression models with additional adjustment for the number of PPE needs and the number of ICP needs. The inclusion of these 2 measures did not meaningfully change the estimates presented in Table 2 (results available from authors on request).

In conclusion, we observed very high levels of depression and anxiety symptoms using validated screening instruments in a sample of health-care workers in Canada during the peak of the COVID-19 pandemic. We also observed important numbers of health-care workers with perceived unmet needs related to both PPE and ICP related to COVID-19 disease transmission. Absolute differences of 18% and 17% were observed in the prevalence of GAD-2 scores of 3 and higher between workers whose perceived PPE and ICP needs were met compared to those who needs were not met. Differences of between 11% and 19% were observed in the prevalence of PHQ-2 scores of 3 and higher across these same PPE and ICP categories. Our results suggest strengthening employer-based infection control strategies could have important implications for mental health symptoms among health-care workers.

## Supplemental Material

Supplemental Material, Smith_Appendix - The Association between the Perceived Adequacy of Workplace Infection Control Procedures and Personal Protective Equipment with Mental Health Symptoms: A Cross-sectional Survey of Canadian Health-care Workers during the COVID-19 Pandemic: L’association entre le caractère adéquat perçu des procédures de contrôle des infections au travail et de l’équipement de protection personnel pour les symptômes de santé mentale. Un sondage transversal des travailleurs de la santé canadiens durant la pandémie COVID-19 Click here for additional data file. Supplemental Material, Smith_Appendix for The Association between the Perceived Adequacy of Workplace Infection Control Procedures and Personal Protective Equipment with Mental Health Symptoms: A Cross-sectional Survey of Canadian Health-care Workers during the COVID-19 Pandemic: L’association entre le caractère adéquat perçu des procédures de contrôle des infections au travail et de l’équipement de protection personnel pour les symptômes de santé mentale. Un sondage transversal des travailleurs de la santé canadiens durant la pandémie COVID-19 by Peter M. Smith, John Oudyk, Guy Potter and Cameron Mustard in The Canadian Journal of Psychiatry
