# Supplementary material for: The Association between the Perceived Adequacy of Workplace Infection Control Procedures and Personal Protective Equipment with Mental Health Symptoms: A Cross-sectional Survey of Canadian Health-care Workers during the COVID-19 Pandemic: L’association entre le caractère adéquat perçu des procédures de contrôle des infections au travail et de l’équipement de protection personnel pour les symptômes de santé mentale. Un sondage transversal des travailleurs de la santé canadiens durant la pandémie COVID-19
Source: Can J Psychiatry. 2020 Sep 21;66(1):17–24. doi: 10.1177/0706743720961729 (PMC7509238; doi:10.1177/0706743720961729)
Supplement: Supplemental Material, Smith_Appendix - The Association between the Perceived Adequacy of Workplace Infection Control Procedures and Personal Protective Equipment with Mental Health Symptoms: A Cross-sectional Survey of Canadian Health-care Workers during the COVID-19 Pandemic: L’association entre l [file Smith_Appendix.pdf]

Table A1: Distribution of study covariates. Canadian healthcare workers (N = 5,988)

Measure	N	Pct of sample*
**Age**		
18 to 24 yrs	202	3.8%
25 to 34 yrs	1 357	25.6%
35 to 44 yrs	1 504	28.4%
45 to 54 yrs	1 319	24.9%
55+ years	917	17.3%
Female	4 792	90.8%
Visible Minority	156	3.0%
**Province of Residence**		
Newfoundland	59	1.1%
Nova Scotia	159	3.0%
New Brunswick	76	1.5%
Ontario	2 950	56.3%
Manitoba	24	0.5%
Saskatchewan	53	1.0%

Alberta	412	7.9%
British Columbia	1 501	28.7%
Other (includes Quebec)	5	0.1%
**Geographic Population Density**		
Urban	3 103	59.0%
Suburban	1 146	21.8%
Rural	1 014	19.3%
**Type of Healthcare facility**		
Hospital	3 429	67.2%
Long-Term Care	752	14.7%
Retirement Home	86	1.7%
Home Care	179	3.5%
Community Care	290	5.7%
Emergency Medical Services	62	1.2%
Public Health	62	1.2%
Other	245	4.8%
**Job Tenure**		
Less than 2 years	852	15.8%

2 to 5 years	1 016	18.8%
5 to 10 years	1 174	21.8%
More than 10 years	2 348	43.6%
**Current working hours**		
None	137	2.5%
1 to 29 hours	823	15.1%
30 to 54 hours	4 062	74.5%
55+ hours	427	7.8%
**Contact with COVID-19 patients**		
Direct contact or within 6 feet	2 302	38.7%
More than 6 feet away/same level/ward/dept	475	8.0%
Visit level/ward/dept or some shared work spaces	1 146	19.2%
No contact respondent aware of	2 032	34.1%
**Number of patients in workplace with COVID-19 (suspected, presumed and confirmed)**		
None	1 165	19.5%
1 to 5	1 079	18.1%
6 to 20	1 149	19.2%
More than 20	1 344	22.5%

Don't know	1 237	20.7%
**Number of co-workers in workplace with COVID-19 (suspected, presumed and confirmed)**		
None	1 918	32.2%
1 to 5	1 231	20.7%
6 to 20	541	9.1%
More than 20	232	3.9%
Don't know	2 036	34.2%
**Experienced COVID-19 symptoms**		
Yes	1 288	21.5%
**Received adequate training for working with COVID-19**		
Yes	1 114	18.6%
**Sufficiently trained to don and doff PPE**		
Yes	3 322	55.8%

* Does not include missing responses
